# Haematological profile abnormalities; and its relationship with severity and outcome of COVID-19 infection

**DOI:** 10.4314/ahs.v22i3.53

**Published:** 2022-09

**Authors:** Noman Ahmed Chaudhary, Muhammad Khurram, Tahira Yasmin, Abdullah Sadiq, Javaria Malik, Qaiser Aziz, Muddesir Nazar, Muhammad Shahzad Manzoor, Muhammad Mujeeb Khan

**Affiliations:** 1 Department of Medicine, Rawalpindi Medical University and Allied Hospitals, Rawalpindi, Pakistan; 2 Department of Infectious Diseases, Rawalpindi Medical University and Allied Hospitals, Rawalpindi, Pakistan

**Keywords:** COVID-19, hematology, corona virus, outcome

## Abstract

**Background:**

Corona virus disease (Covid-19) caused by corona virus (SARS Cov-2) has affected millions of people around the world. Many diagnostic modalities have been tested but the blood complete picture remains the initial and most easily accessible investigation in Covid-19.

**Objectives:**

The objective of this study was to find out the haematological abnormalities in relation to Covid-19 severity and outcome.

**Methods:**

This cross-sectional study was carried out from April 2020 to July 2020. One--hundred and fifty polymerase chain reaction (PCR) confirmed Covid-19 patients were inducted by random sampling. Haematological profile at admission was recorded. Data thus obtained was analyzed with respect to Covid-19 severity and outcome. The data was entered and analyzed using Statistical Package for Social Sciences (SPSS) version 19.

**Results:**

Out of a total of 150 patients included in the study, 77(51.3%) patients had mild disease at the time of admission, 42 (28%) had moderate disease while 31 (20.7%) had critical disease at the time of admission. Medians (interquartile range) of total leucocyte count (TLC), neutrophils, lymphocytes, neutrophils to lymphocytes ratio (NLR), platelets to lymphocytes ratio (PLR), neutrophils to monocyte ratio (NMR), monocyte to lymphocyte ratio (MLR) were 8.11 (IQR=4.88), 5.95 (IQR=4.58), 1.66 (IQR=1.10), 3.48 (IQR=4.20), 146.24 (IQR=130.75), 18.87 (IQR=14.07), 0.16 (IQR=0.13). Median NLR was higher in patients with critical illness 11.23 (IQR=10.70) as compared to those with stable 2.51 (IQR=1.77) and moderate 3.22 (IQR=3.60) disease (p< 0.000). Similarly TLC (p< 0.000), neutrophils (p< 0.000), lymphocytes (p< 0.000), NLR (p< 0.000), PLR (p< 0.000, p=0.001), MLR (p< 0.000), NMR (p< 0.000) had significant relationship with the severity and outcome of Covid-19 infection.

**Conclusion:**

Many haematological parameters are significantly different and can be used to predict the severity and outcome of Covid-19 infection.

## Introduction

Severe acute respiratory syndrome corona virus 2 (SARS-CoV-2) causing Corona virus disease 2019 (COVID-19) turned from an epidemic outbreak, into a pandemic affecting millions of individuals all over the world^1^. The disease emerged in December 2019, from Wuhan, Hubei, China as a respiratory disease with a clinical presentation resembling viral pneumonia^2^. Analysis of samples from lower respiratory tract showed these to be enveloped, non-segmented positive sense RNA viruses belonging to the family Corona viridae and the order Nidovirales^3^. COVID-19, established as an infection of the respiratory tract, is now suggested by recent data to be regarded as a multi-systemic disease involving cardiovascular, respiratory, gastrointestinal, neurological, hematopoietic and immune system^4^–^6^. Most of the people infected with COVID-19 have mild to moderate symptoms recovering after proper interventions. However, some patients develop severe disease. The case-fatality rate tends to go higher with the severity of disease and increase in age of the patients^7^.

Typically, hematological profile is one of the first test requested by a physician in Covid-19 infection as in most of the other diseases. A complete blood count (CBC), reporting also a white blood cell (WBC) differential, can be a both rapid and cost-efficient tool for detection of viral infections^8^. Many haematological abnormalities are associated with Covid-19 infection. A number of studies have shown that higher Total leukocyte count (TLC) and neutrophils, lymphocytopenia and thrombocytopenia are associated with severe Covid-19 and worst outcomes^9^. Worldwide more than 14 million cases have been affected with 654 000 deaths up to 22nd July 2020^10^. In Pakistan, to date 256605 cases and 5709 deaths have been reported^11^. The actual number of cases might exceed the number reported due to asymptomatic and under-reported mild disease. Rawalpindi Medical University attached hospitals were the hub of Covid-19 management. More than 1200 cases of Covid-19 in Rawalpindi Division of Pakistan were managed at these hospitals from March 2020 to July 2020. This study aimed to describe and analyze the hematological abnormalities of confirmed cases of COVID-19 and their relation with the severity and outcome of the disease in these patients.

## Methods

This prospective cross-sectional study was carried out at Rawalpindi Medical University attached hospitals, from April 2020 to July 2020. After the approval from the Institutional Research Forum of the university. Random sampling technique was used and 150 patients were selected from a cohort of 1119 patients. WHO sample size calculator was used to calculate the sample size by using the formula (n= Z2*p*(1-p)/d2). Approximate positivity rate of new COVID-19 infections was 9% during April 2020. Margin of error was 5% and confidence level was 95%. Lottery method was used to select the patients for the study. Patients with clinical symptoms of Covid-19 and a confirmed positive reverse transcriptase-polymerase chain reaction (RTPCR) of nasal and pharyngeal swabs were included in the study. Consent was taken from the patients or and their surrogates and patient confidentiality was maintained.

The severity of COVID-19 was graded as stable, moderate and critical. Stable patients included those who had minor symptoms and no imaging evidence of pneumonia, those having fever, respiratory tract symptoms, and pneumonia on imaging. Patients having moderate disease had respiratory distress, respiratory rate ≥ 30 beats/minute, mean oxygen saturation ≤ 93% in resting state, arterial blood oxygen partial pressure/oxygen concentration ≤ 300mmHg (1mmHg = 0.133kPa) and pulmonary imaging showing more than 50% lung involvement within 24–48 hours. Critical patients were those who had one of the following conditions: respiratory failure requiring mechanical ventilation, Shock, ICU admission for combined organ failure. The severity was graded using National Institute of Health Guidelines^12^.

For each patient blood complete picture was ordered at admission. Report was generated by automated blood analyzer. Patients were managed according to the national and international guidelines and were followed until recovery and discharge or death. Haematological parameters like total leukocyte count (TLC), neutrophil, lymphocytes, monocytes, platelets, red blood cells (RBCs), haemoglobin, mean corpuscular volume (MCV), mean platelet volume (MPV), haematocrit (HCT), plateletcrit (PCT), red cell distribution width (RDW), and platelet distribution width (PDW) were compared across age groups, gender, severity of Covid-19, outcome in terms of in-hospital mortality or discharge.

Data was entered and analyzed using Statistical Package for Social Sciences (SPSS) version 25. Qualitative variables like age groups, gender, outcome, severity of Covid-19 and comorbids were represented as frequencies and percentages. Independent samples Kruskal-Wallis tests, Mann Whitney U test and ANOVA were applied for comparing quantitative variables across categories of qualitative variables. Receiver operator curves (ROC) were obtained for detecting the cut off points of significant haematological parameters to predict outcome and severity of the disease. Stepwise multinominal logistic regression was conducted to find significant predictors of severity and outcome of Covid-19. A p-value of ≤ 0.05 was considered significant.

## Results

Out of a total 150 patients, 85 (56.7%) were males whereas 65 (43.3%) were females. Mean age of the patients was 48.9 ± 17.8 years. One hundred and thirty-eight (92%) patients had no travel history in the past 14 days while 12 (8%) had travelled abroad in the past two weeks. Eighty-one (54%) patients had no comorbids while 69 (46%) had history of comorbids. Seventy-seven (51.3%) patients had mild disease at the time of admission, 42 (28%) had moderate disease while 31 (20.7%) had critical disease at the time of admission. Out of 150 patients 20 (13.3%) patients died in the hospital. Median time of hospital stay was 16 (IQR= 9) days.

The severity of Covid-19 was not statistically significant between males and females (p= 0.363). Mean age of the stable patients was 42.1 ± 16.67 years, that of the moderate patients was 49.28 ± 14.83 years and critical patients was 65.26 ± 13.39 years. The results of one-way ANOVA showed that mean age was statistically significant among stable, moderate and critically ill patients (p< 0.000). The severity of Covid-19 was also significantly related to the presence of comorbids (p< 0.000). Most of the patients who died in the hospital also had one or more comorbidities as compared to those who were discharged (p< 0.000). Mean age of the non-survivors was also significantly higher as compared to survivors (p< 0.000). This is shown in Table 1.

**Table 1:** Table showing relationship between severity of COVID-19 with gender, presence of comorbids and age

Parameter	Groups	Severity			P-vlaue	Outcome		p-value
		Mild	Moderate to severe	Critical		Discharged	Dead	
Gender	Male	47 (55.3%)	20 (23.5%)	18 (21.2%)	0.363*	73 (85.9%)	12 (14.1%)	0.747*
	Female	30 (46.2%)	22 (33.8%)	13 (20%)		57 (87.7%)	8 (12.3%)	
Comorbids	Yes	21 (30.4%)	22 (31.9%)	26 (37.7%)	**< 0.000** *	52 (75.4%)	17 (24.6%)	**< 0.000** *
	No	56 (69.1%)	20 (24.7%)	5 (6.2%)		78 (96.3%)	3 (3.7%)	
Age	Mean ± SD	42.12 ± 16.67	49.28 ± 14.83	65.26 ± 13.39	**< 0.000** **	46.32 ± 17.16	65.7 ± 12.23	**< 0.000** **
Days in hospital	Median (IQR)	17 (8)	13 (6)	17 (14)	**0.016** **	16 (7)	16.5 (15.5)	0.916**

* Chi-square test

** One-way Analysis of Variance

*** Kruskal Wallis test

Out of the 77 patients with stable and 42 patients with moderate disease none died in the hospital whereas out of 31 patients with critical disease 20 (64.5%) died in the hospital (p< 0.000)

TLC and neutrophil count was significantly higher in the patients aged 60 and above (p= 0.006, p< 0.000) respectively while lymphocyte count was significantly lower (p= 0.001). Similarly, NLR (p< 0.000), PLR (p= 0.005) and MLR (p< 0.000) had statistically significant relationship with age Medians of blood counts along-with other haematological parameters and their relationship with age and gender is shown in Table 2.

**Table 2:** Table showing comparison of haematological parameters between gender and age groups.

Haematological profile	Total median (IQR)	Gender			Age groups		
		Male median (IQR)	Female median (IQR)	p- value*	≤ 40 years median (IQR)	41–60 years median (IQR)	≥ 60 years median (IQR)	p- value**
Total leukocyte count	8.11 (4.88)	8.21 (4.99)	8.09 (4.62)	0.703	7.74 (2.83)	8.32 (4.78)	9.90 (6.48)	**0.006**
Neutrophils	5.95 (4.58)	6.12 (4.69)	5.72 (4.32)	0.241	4.88 (2.82)	6.12 (4.03)	7.63 (7.01)	**<0.000**
Lymphocytes	1.66 (1.10)	1.56 (0.95)	1.92 (1.23)	**0.002**	2.01 (0.95)	1.61 (1.06)	1.28 (0.97)	**0.001**
Monocytes	0.30 (0.18)	0.28 (0.17)	0.32 (0.22)	**0.068**	0.31 (0.14)	0.30 (0.24)	0.28 (0.20)	0.886
Platelets	246.50 (149.50)	243.00 (146.75)	248.00 (157.25)	0.900	246.00 (149.00)	259.00 (174.50)	242.00 (115.75)	0.834
Red blood cells	4.72 (0.76)	4.90 (0.71)	4.43 (0.78)	**< 0.000**	4.75 (0.63)	4.82 (0.66)	4.42 (0.86)	0.018
Haemoglobin	13.40 (2.80)	14.5 (1.80)	12.15 (2.35)	**< 0.000**	13.80 (3.00)	13.70 (2.65)	12.60 (3.32)	0.002
Haematocrit	41.60 (8.65)	44.15 (6.43)	37.10 (7.13)	**< 0.000**	42.80 (10.80)	42.60 (7.80)	39.15 (9.23)	0.013
Plateletcrit	0.20 (0.13)	0.21 (0.13)	0.20 (0.12)	0.764	0.20 (0.13)	0.22 (0.16)	0.19 (0.09)	0.757
RDW	11.20 (1.60)	10.90 (1.17)	11.45 (1.67)	**0.012**	11.10 (1.90)	11.10 (1.60)	11.50 (1.47)	0.192
PDW	8.85 (1.80)	8.80 (1.47)	8.90 (2.28)	0.824	8.80 (2.30)	9.10 (1.70)	8.85 (1.90)	0.329
MCV	89.70 (7.75)	90.80 (7.22)	86.95 (8.75)	**< 0.000**	90.40 (5.90)	89.50 (8.60)	86.55 (8.92)	0.101
MPV	8.55 (0.90)	8.60 (0.90)	8.50 (1.00)	0.683	8.30 (1.00)	8.60 (0.85)	8.40 (1.27)	0.338
NLR	3.48 (4.20)	3.89 (6.44)	2.67 (3.37)	**0.016**	2.45 (1.78)	3.56 (3.84)	5.40 (8.73)	**< 0.000**
PLR	146.24 (130.75)	158.30 (141.33)	131.43 (125.69)	0.020	124.16 (73.27)	163.92 (143.58)	183.65 (151.47)	**0.005**
MLR	0.16 (0.13)	0.17 (0.13)	0.16 (0.11)	0.190	0.15 (0.05)	0.16 (0.21)	0.22 (0.16)	**< 0.000**
NMR	18.87 (14.07)	20.53 (24.23)	18.17 (10.72)	**0.041**	17.50 (8.82)	19.74 (17.23)	25.52 (25.69)	0.016
HCT to PCT ratio	188.68 (134.69)	205.28 (138.42)	181.67 (111.04)	0.060	188.46 (120.23)	194.40 (161.33)	181.92 (96.96)	0.731

* Mann Whitney U test

** Kruskal Wallis test

RDW; Red Cell Distribution Width, PDW; Platelet distribution width, MCV; Mean Corpuscular Volume, MPV; Mean Platelet Volume, NLR; Neutrophil to Lymphocyte Ratio, PLR; Platelet to Lymphocyte ratio, MLR; Monocyte to Lymphocyte ratio, NMR; Neutrophil to Monocyte ratio, HCT; Haematocrit, PCT; Plateletcrit

The results of independent samples Kruskal-Wallis test showed that the medians of total leukocyte count (TLC), neutrophil count, lymphocyte count, platelet count, red blood count, haematocrit and plateletcrit, neutrophil to lymphocyte ratio (NLR), platelet to lymphocyte ratio (PLR), neutrophil to monocyte ratio (NMR), monocyte to lymphocyte ratio (MLR) and haematocrit to platelet-crit ratio (HCT to PCT ratio) were significantly different among the patients with stable, moderate and critical illness. TLC, neutrophils, lymphocytes, platelets, plateletcrit, NLR, PLR, MLR, NMR and HCT to PCT ratio were also significantly different between the patient with in-hospital mortality and those who were discharged. This is shown in Table 3.

**Table 3:** Table showing comparison of haematological parameters according to severity and outcome of Covid-19 patients

Haematological profile	Severity			P-value*	Outcome		p-value**
	Mild median (IQR)	Moderate to severe median (IQR)	Critical median (IQR)		Discharged median (IQR)	Dead median (IQR)	
Total leukocyte count (TLC)	7.20 (3.01)	9.01 (3.92)	13.02 (6.92)	**< 0.000**	0.82 (0.91)	13.36 (6.41)	**< 0.000**
Neutrophils	4.84 (2.27)	6.71 (4.26)	11.14 (6.60)	**< 0.000**	5.52 (0.47)	12.14 (5.37)	**< 0.000**
Lymphocytes	1.70 (0.94)	2.00 (0.78)	1.01 (0.53)	**< 0.000**	1.73 (0.97)	0.82 (0.49)	**< 0.000**
Monocytes	0.28 (0.17)	0.35 (0.27)	0.29 (0.21)	0.138	0.30 (0.18)	0.29 (0.22)	0.615
Platelets	270.00 (145.75)	224.00 (168.00)	202.00 (135.00)	**0.001**	257.50 (151.50)	194.00 (150.25)	**0.022**
Red blood cells	4.83 (0.63)	4.47 (0.71)	4.45 (0.95)	**0.006**	4.73 (0.72)	4.46 (1.04)	0.288
Haemoglobin	14.05 (2.85)	13.00 (2.30)	13.00 (3.20)	0.054	13.40 (2.78)	13.05 (3.22)	0.412
Haematocrit	43.45 (7.88)	39.90 (7.35)	38.80 (9.30)	**0.001**	42.25 (8.38)	38.95 (10.42)	0.082
Plateletcrit	0.24 (0.12)	0.17 (0.13)	0.17 (0.12)	**0.001**	0.22 (0.13)	0.16 (0.12)	**0.012**
RDW	11.05 (1.57)	11.30 (1.60)	11.70 (1.60)	0.108	11.15 (1.60)	11.55 (1.55)	0.204
PDW	8.80 (1.85)	9.00 (1.90)	8.70 (2.40)	0.355	8.85 (1.80)	8.80 (2.65)	0.145
MCV	89.9 (9.13)	90.30 (8.30)	86.60 (8.50)	0.145	89.80 (7.57)	86.50 (11.92)	0.127
MPV	8.60 (0.90)	8.60 (0.95)	8.30 (1.30)	0.462	8.6 (1.00)	8.30 (0.75)	0.092
NLR	2.51 (1.77)	3.22 (3.60)	11.23 (10.70)	**< 0.000**	2.87 (3.13)	13.28 (7.21)	**< 0.000**
PLR	144.56 (115.44)	113.50 (116.47)	237.86 (195.44)	**< 0.000**	143.72 (107.70)	259.34 (186.84)	**0.001**
MLR	0.15 (0.07)	0.16 (0.13)	0.33 (0.30)	**< 0.000**	0.15 (0.11)	0.36 (0.36)	**< 0.000**
NMR	16.95 (6.15)	18.50 (18.54)	31.59 (28.36)	**< 0.000**	18.14 (11.88)	32.18 (34.04)	**< 0.000**
HCT to PCT ratio	180.36 (110.77)	215.79 (162.58)	238.12 (167.93)	**0.012**	182.12 (121.20)	261.43 (168.17)	**0.031**

* Kruskal Wallis test

** Mann Whitney U test

RDW; Red Cell Distribution Width, PDW; Platelet distribution width, MCV; Mean Corpuscular Volume, MPV; Mean Platelet Volume, NLR; Neutrophil to Lymphocyte Ratio, PLR; Platelet to Lymphocyte ratio, MLR; Monocyte to Lymphocyte ratio, NMR; Neutrophil to Monocyte ratio, HCT; Haematocrit, PCT; Plateletcrit

The results of step-wise multinominal logistic regression show that NLR, PLR, haemoglobin monocyte count age and presence of comorbids are the best predictors of severity of COVID-19 as shown in Table 4

**Table 4:** Table showing results of stepwise multinominal logistic regression for predicting severity of Covid-19

Severity	Predictors	B	Exp (B)	p-value	Confidence interval (95%)
Stable	Age	-0.052	0.950	0.070	0.898–1.004
	Haemoglobin	0.456	1.578	**0.028**	1.051–2.369
	NLR	-0.908	0.403	**< 0.000**	0.280–0.581
	PLR	0.013	1.013	**0.009**	1.003–1.023
	Monocyte	-4.508	0.011	**0.029**	<0.000–0.635
	Comorbids	-2.384	0.092	**0.014**	0.014–0.614
Moderate	Age	-0.042	0.959	0.108	0.911–1.009
	Haemoglobin	0.111	1.117	0.499	0.810–1.541
	NLR	-0.270	0.764	**0.005**	0.632–0.922
	PLR	-0.002	0.998	0.520	0.990–1.005
	Monocyte	-0.724	0.485	0.648	0.022–10.826
	Comorbids	-1.698	0.183	0.053	0.033–1.022

RBC; Red Blood Cells, NLR; Neutrophil to Lymphocyte ratio, PLR; Platelet to Lymphocyte ratio

The results of stepwise Cox regression showed that the model containing age (B= 0.057, Exp B= 1.058, p= 0.006), NLR (B= 0.122, Exp B= 1.129, p< 0.000) and PDW (B= - 0.356, Exp B= 0.694, p= 0.002) were the best predictors for in-hospital mortality from Covid-19.

The area under curve (AUC), cut-off values, sensitivity and specificity of different haematological parameters is shown as follows.

**Figure 1:**
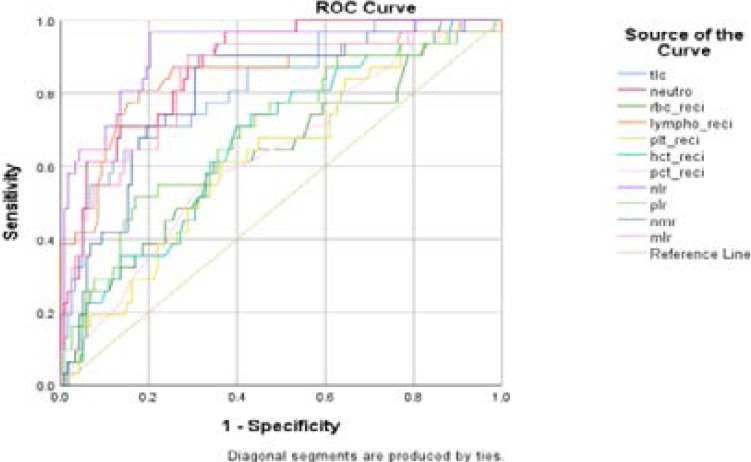
ROC curve showing different haematological markers for predicting the severity of Covid-19.

**Figure 2:**
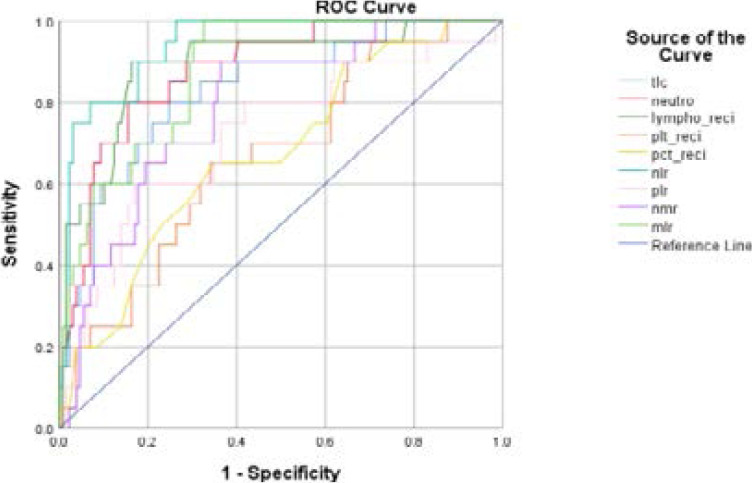
ROC curve showing different haematological markers for predicting the outcome of Covid-19.

The ROC curves of different haematological markers in predicting the severity and outcome of Covid-19 are shown in figure 1 and figure 2 respectively

## Discussion

Most of the patients in our country were of mild to moderate severity similar to those in China^13^. Our study showed that 20.7% of the patients had critical disease at the time of admission. Most of the patients with critical disease died in the hospital in our study. Another study showed that those who are severely affected by the disease show high mortality rates. So, it is important to predict the severity of the disease on admission for better management and to reduce the mortality associated with severe disease^14^.

Our study showed that the mean age of the patients with critical disease was higher 65.26 ± 13.39 as compared to those with mild to moderate disease. Wang et al. also showed that the median age of the patients with critical disease was 65 years^15^. The mean age of the patients with in-hospital mortality was also higher as compared to those who were discharged with negative PCR. This indicates that age is a significant indicator of severity and mortality from Covid-19 as mentioned by another study also^16^.

Our study also showed that the presence of comorbidities was also significantly related to severe disease and in-hospital mortality. Other studies also supported this by reporting higher prevalence of comorbidities in critical patients and those who died in a health-care facility^14^,^15^. These results show that presence of comorbidities may also point towards poorer prognosis and outcome of Covid-19^15^.

Our study showed that several haematological markers can be used for predicting the severity and outcome of Covid-19. Total leukocyte count, neutrophil count, lymphocytes were significantly different in the three subgroups of severity. Patients with critical disease had higher TLC and neutrophil count and lower lymphocyte count as compared to those with stable and moderate disease. The results by Liu et al. and Wan et al. also confirmed that neutrophil count and lymphocyte count were related to severity of the disease^16^,^17^. The patients who died in the hospital also had significantly raised TLC and neutrophil count and decreased lymphocyte count as compared to those who were discharged from the hospital. The study by Wang et al. confirmed that increased neutrophil count is associated with poorer outcomes in patients with Covid-19^18^. According to a study increased TLC is associated with an increased severity of systemic inflammatory response as compared to those with normal TLC^19^. This leads to neutrophil migration, recruitment and activation in the pulmonary vasculature through the production of several interleukins. Although this mechanism is intended for protection against the virus, higher neutrophil count causes tissue damage causing increased severity of pneumonia and death^19^,^20^. The reasons for lymphocytopenia include 1) direct infection and destruction of the lymphocytes by the virus^21^. 2) destruction of the lymphocytic organs like spleen and thymus by the virus^22^. 3) lymphocyte destruction by the cytokine storm which includes tumour necrosis factor (TNF)α, interleukin (IL)-6, and other pro-inflammatory cytokines^23^. 4) Lymphocyte inhibition due to metabolic side-products like lactic acid produced during sepsis^24^.

We also found some new haematological parameters, which were related to the severity and outcome of the disease these were Red blood cell counts, platelets, haematocrit, plateletcrit. The patients with critical disease and those who died in the hospital had significantly lower levels of these parameters as compared to those who had stable and moderate disease. Patients who suffered in-hospital mortality also had significantly lower levels of these parameters as compared to those who were discharged from the hospital. The study by Liu et al. also showed the critical patients had lower levels of platelets (153.0 x 10^9/L) but the results were not significant^25^. Nasiri et al. also found that 11.1% of the patients had thrombocytopenia^26^. Other studies by Lippi et al. and Huang et al. also reported that thrombocytopenia was associated with sever disease in Covid-19^27^,^28^ . This shows that these parameters can also be used to predict the severity and mortality of Covid-19. Many mechanisms have been proposed in this regard. These include 1) destruction of hematopoietic stem cells by cytokine storm 2) direct infection of the hematopoietic stem cells by the virus 3) formation of autoantibodies and immune complexes against the platelets 4) increased platelet activation and aggregation in pulmonary bed causing decrease in circulating platelets^29^. Similarly due to relatively lower number of platelets, plateletcrit was also significantly lower in critical disease. Although red blood cell count, platelet count and plateletcrit were in the normal range in mild, moderate to severe and critical disease to be labelled as anaemic or thrombocytopenic yet the decreasing trend may be used to predict the severity of the disease. This can be irrespective of the values used as a cut off for anaemia and thrombocytopenia.

In our study patients with severe disease had significantly higher neutrophil to lymphocyte ratio, platelet to lymphocyte ratio, monocyte to lymphocyte ratio and monocyte to neutrophil ratio as compared to stable and moderate disease. These parameters were also significantly higher in the patients who died in the hospital. In the study by Yang et al. NLR (20.7 ± 24.1 vs. 4.8 ± 3.5), PLR (436.5 ± 329.2 vs. 176.7 ± 84.2) and LMR (2.1 ± 1.6 vs. 4.1 ± 6.0) were significantly different between critical and non-critical patients^30^.

Our study showed that NLR was the best diagnostic marker for predicting the severity with an AUC of 0.917 followed by neutrophil count, reciprocal of lymphocyte count, MLR, TLC and NMR. We have observed higher sensitivity and specificity respectively of NLR (87.1%, 80.7%) and PLR (74.2%, 56.3%) in diagnosing the severity as compare to those by Yang et al., 63.6% ,88% for NLR and 44% and 77% for PLR^30^.

In our study patients who died in hospital had significantly higher median NLR as compared to those who were discharged (13.28 vs. 2.87). In the study by Yan et al. non-survivors had a high NLR (49.06) as compared to those who survived (4.11)^31^. Similarly, patients who died in the hospital had significantly raised PLR, NMR, MLR and haematocrit to plateletcrit ratio as compared to survivors. These new parameters could also be used to predict mortality in the patients with Covid-19.

Our study shows that simple haematological parameters can be of value for predicting the severity of Covid-19 infection in resourse limited and overburdened settings. This study was conducted in a single tertiary care centre which is a limitation of this study. Conducting it in multiple units using a large cohort will help in better understanding and analysis of the disease characteristics. Another possible limitation is that the effect of comorbids on the haematological parameters couldn't be controlled. Further studies conductd by eliminating the effect of comorbids should be conducted.

## Conclusion

Age and presence of comorbids predict the severity and outcome of Covid-19. Older patients and those with one or more premorbids are more prone to critical illness and are more likely to die in the hospital as compare to the young and without any comorbids. Total leukocyte count, neutrophils, lymphocytes, platelets, Red blood cells, haematocrit, platletcrit, NLR, PLR, NMR, MLR and HCT to PCT ratio were significantly different between the patients with stable, moderate and critical disease. Total leukocyte count, neutrophils, lymphocytes, platelets, plateletcrit, NLR, PLR, NMR, MLR and HCT to PCT ratio were also significantly different between survivors and non-survivors. Age, comorbids, haemoglobin, NLR, PLR and monocyte count are the best predictors of critical illness. While age, PDW and NLR are best predictors of in-hospital mortality.

## Figures and Tables

**Table 5:** Table showing area under curve (AUC), cut-off values, sensitivity and specificity of different haematological parameters for detecting severity (critical) and outcome (mortality) patients

Parameter	Area under curve	p-value	Cut-off value	Sensitivity	Specificity
Severity					
TLC	0.819	< 0.000	11.295	64.5%	85.7%
Neutrophils	0.876	< 0.000	7.095	80.6%	74.8%
Lymphocytes	0.847	< 0.000	1.31	80.6%	82.4%
Platelets	0.620	0.039	222.22	61.3%	63%
RBC	0.638	0.018	4.53	61.3%	65.5%
HCT	0.670	0.004	40.48	64.5%	61.9%
PCT	0.632	0.024	0.19	61.3%	56.8%
NLR	0.917	< 0.000	5.12	87.1%	80.7%
PLR	0.699	0.001	145.80	74.2%	56.3%
NMR	0.804	< 0.000	20.77	87.1%	69.7%
MLR	0.838	< 0.000	0.1951	80.6%	73.9%
Outcome					
TLC	0.832	< 0.000	13.00	60%	90.8%
Neutrophils	0.880	< 0.000	9.12	80%	84.6%
Lymphocytes	0.891	< 0.000	0.98	60%	90%
Platelets	0.659	0.022	243.9	70%	56.2%
PCT	0.674	0.013	0.23	75%	42.6%
NLR	0.939	< 0.000	5.83	90%	82.3%
PLR	0.723	0.001	172	70%	63.8%
NMR	0.788	< 0.000	21.54	75%	65.4%
MLR	0.878	< 0.000	0.195	90%	70.8%

TLC; Total Leukocyte Count, RBC; Red Blood Cells, HCT; Haematocrit, PCT; Plateletcrit, RDW; Red Cell Distribution Width, NLR; Neutrophil to Lymphocyte Ratio, PLR; Platelet to Lymphocyte ratio, MLR; Monocyte to Lymphocyte ratio, NMR; Neutrophil to Monocyte ratio
